# The clinical potential of antiangiogenic fragments of extracellular matrix proteins

**DOI:** 10.1038/sj.bjc.6602820

**Published:** 2005-10-18

**Authors:** A R Clamp, G C Jayson

**Affiliations:** 1 Cancer Research UK Department of Medical Oncology, Christie Hospital, Wilmslow Road, Manchester M20 4BX, UK

## Abstract

Neovasculature development is a crucial step in the natural history of a cancer. While much emphasis has been placed on proangiogenic growth factors such as VEGF, it is clear that endogenous angiogenesis inhibitors also have critical roles in the regulation of this process. Recent research has identified several cryptic fragments of extracellular matrix/vascular basement membrane proteins that have potent antiangiogenic properties *in vivo*. It has become apparent that many of these fragments signal via interactions with endothelial integrins, although multiple downstream effector pathways have been implicated and endostatin, the first non-collagenous domain of collagen XVIII, influences an intricate signalling network. The activity of these molecules in animal models suggests that they may have significant clinical activity; however, results of phase I/II trials with endostatin were disappointing. Many possible reasons can be found for the failure of these studies. Weaknesses in trial design, endostatin administration regimen and patient selection are identifiable, and importantly the lack of a clearly defined antiangiogenic mechanism for endostatin hindered assessment of biologically effective dose. Additionally, *in vivo* immunological and proteolytic function-neutralising mechanisms may have negated endostatin's actions. Lessons learned from these studies will aid the future clinical development of other antiangiogenic extracellular matrix protein fragments.

Angiogenesis, the development of a neovasculature from pre-existing blood vessels, is vital for tumour growth and metastasis. Without a blood supply, neoplastic deposits remain dependent on diffusion for nutrients, oxygenation and the removal of waste metabolites. Their growth is restricted to a maximum of 1–2 mm^3^ and most remain clinically quiescent.

Interactions between endothelial cells and the extracellular matrix (ECM), in particular components of the vascular basement membrane (VBM), play key roles in the regulation of angiogenesis (Kalluri, 2003). The VBM is constructed from interacting but independent scaffolds of laminins and collagen IV complemented by many other important but less abundant molecules such as perlecan, the fibulins and collagens XV and XVIII. These molecules contribute to the control of endothelial cell development, proliferation, migration and function via interactions with transmembrane signalling molecules such as the integrins and syndecans.

While tumour angiogenesis is characterised by the secretion of a multiplicity of proangiogenic factors tripping the angiogenic switch resulting in the development of a structurally and functionally abnormal vasculature, physiological angiogenesis is tightly controlled and, in most tissues, proangiogenic factors are balanced by endogenous antiangiogenic signals holding the angiogenic switch ‘off’ (Carmeliet and Jain, 2000).

Recently, several ECM/VBM protein fragments have been isolated that have potent antiangiogenic properties that are only apparent after proteolytic cleavage of the fragments from their parental molecule. These cryptic endogenous angiogenesis inhibitors (Nyberg *et al*, 2005) specifically inhibit endothelial cell proliferation and migration *in vitro* and also have impressive antitumour activity *in vivo* (O'Reilly *et al*, 1997; Hamano *et al*, 2003; Bix *et al*, 2004). These findings led to considerable enthusiasm for the clinical investigation of the fragments. However, this excitement has subsequently been tempered by the results obtained in early phase trials of endostatin, a C-terminal proteolytic fragment derived from the first non-collagenous (NC1) domain of collagen XVIII (O'Reilly *et al*, 1997).

In this review, we will briefly describe the antiangiogenic fragments of ECM proteins and then discuss the endostatin trials focusing on what lessons can be learnt to aid us in the clinical development of other endogenous angiogenesis inhibitors.

## MECHANISMS OF ACTION – SIMILARITIES AND DIFFERENCES

Since Homandberg *et al* (1985) isolated 29 and 40 kDa fibronectin fragments that could inhibit endothelial cell proliferation (Homandberg *et al*, 1985), a substantial number of antiangiogenic ECM protein fragments have been identified and these are summarised in Table 1.

The most thoroughly investigated group of ECM fragments are those derived from the first non-collagenous (NC1) domains of VBM collagens – in particular, endostatin (O'Reilly *et al*, 1997) and tumstatin (Maeshima *et al*, 2001; Hamano *et al*, 2003), derived from the *α*1 chain of collagen XVIII and *α*3 chain of collagen IV, respectively. These globular, relatively protease-resistant domains are released from the VBM by the action of proteolytic enzymes at a protease-sensitive hinge region. While several proteases, including elastase and cathepsin L, have been shown to induce endostatin release *in vitro*, matrix metalloprotease-9 (MMP-9) appears to generate tumstatin *in vivo*, as MMP-9 knockout mice have lower circulating levels of tumstatin. Tumour xenografts exhibit rapid growth with increased angiogenesis in these mice in a manner that can be prevented by restoring ‘physiological’ serum tumstatin levels (Hamano *et al*, 2003).

Recent research has identified that integrins on the endothelial cell surface act as signal transducing receptors for many ECM proteolytic fragments (Table 1). Endothelial cells express multiple integrins but *α*v*β*3 and *α*5*β*1 are substantially upregulated in activated endothelia and antagonists of these transmembrane receptors can suppress angiogenesis *in vivo* (summarised in Jin and Varner, 2004). Tumstatin binds *α*v*β*3 integrin and endostatin binds *α*5*β*1, resulting in inhibition of downstream focal adhesion kinase activation (Sudhakar *et al*, 2003) although the antiangiogenic effector pathways are distinct for each molecule. Tumstatin inhibits CAP-dependent protein translation via phosphatidylinositol-3 kinase (PI3K)/Akt/mTOR and 4E-BP1, resulting in endothelial cell apoptosis (Maeshima *et al*, 2001; Sudhakar *et al*, 2003). Endostatin, however, modulates focal adhesion and actin stress fibre formation. In human dermal microvascular cells (HDMECs), endostatin induces clustering of *α*5*β*1 integrins within lipid raft microdomains in a manner that is dependent on cell surface heparan sulphate proteoglycans (HSPGs). This clustering activates Src kinases that downregulate RhoA activity, so dissociating focal adhesions and actin stress fibres and inhibiting endothelial cell migration (Wickström *et al*, 2003). Notably, the addition of a second integrin-binding site to the N-terminal of endostatin appears to increase its antiangiogenic potency (Yokoyama and Ramakrishnan, 2004).

While this signalling pathway for endostatin has been well elucidated, multiple other potential antiangiogenic mechanisms and ligands for endostatin have been described. These include direct interaction with cell surface KDR (Kim *et al*, 2002) and glypican HSPGs (Karumanchi *et al*, 2001), binding to tropomyosin isoform 3 (MacDonald *et al*, 2001), inhibition of MMP-2 (Lee *et al*, 2002) and downregulation of *β*-catenin via Wnt signalling pathways (Hanai *et al*, 2002). Notably, examination of the downstream effects of endostatin in HDMECs by gene expression profiling and phosphoproteomics (Abdollahi *et al*, 2004) detected the coordinated regulation of eight angiogenic effector pathways with the expression of 12% of assessed genes being altered at least two-fold after 2 h endostatin exposure. Although this investigation gives us just a snapshot of the *in vitro* effects of a fixed concentration of endostatin at a single time point in one endothelial cell line, it demonstrates that endostatin influences a remarkable and intricate signalling network in order to assert its antiangiogenic effects. Clearly, further work needs to be performed to validate the data and explore what factors allow endostatin to target tumour endothelial cells specifically in animal models without adversely affecting physiological angiogenesis. Similar investigations with other ECM angiogenesis inhibitors may identify further similarities or differences to endostatin, which could direct the development of combination antiangiogenic approaches.

## A PHYSIOLOGICAL ROLE?

Although a substantial body of evidence now exists to support an antiangiogenic role for pharmacological doses of ECM fragments in experimental systems, data indicating a physiological role for these molecules are scarce. Endostatin is present at detectable levels in the serum of normal individuals (Feldman *et al*, 2002; IIzasa *et al*, 2004) and canstatin/arresten has been isolated from human placenta, suggesting physiological function. Further indirect evidence is provided by the study of mouse strains with null mutations in the parental ECM molecule and rare single gene disorders in humans (see Table 2). While these models implicate ECM fragments such as endostatin and restin in vascular development in specific organs, it appears that in the majority of tissues there is ‘physiological redundancy’ with the absence of a single fragment compensated for by other proteins.

Importantly however, Sund *et al* (2005) recently presented evidence suggesting that tumstatin, endostatin and thrombospondin-1 have endogenous tumour suppressor functions. Using tumstatin- and endostatin-deficient mouse strains, they documented accelerated growth of Lewis lung carcinoma/B16F10 melanoma xenografts compared to identical tumours in the parental mouse lines. Notably, tumour growth was even more rapid in mice deficient in both tumstatin and thrombospondin-1, indicating that these three molecules suppress the growth of tumour xenografts when present at physiological concentrations. This observation was reinforced by the observation that B16F10 xenograft growth was suppressed in a mouse line engineered to overexpress endostatin resulting in a 1.6-fold increase in circulating endostatin levels. This latter model recapitulates the clinical observation that patients with Down's syndrome (trisomy 21) who have three copies of the collagen XVIII gene and 70% higher serum endostatin levels (Zorick *et al*, 2001) have much lower standardised mortality odds ratios for death from solid malignant tumours at all ages than the rest of the population (Yang *et al*, 2002), suggesting that endostatin may have an endothelium-specific tumour suppressor function in humans.

## CLINICAL STUDIES

While most antiangiogenic ECM fragments have yet to enter clinical development, three phase I trials have been published using recombinant human endostatin in a total of 61 patients with advanced metastatic cancer (including 11 melanoma, 10 sarcoma, nine colorectal, nine lung, seven breast, four renal) (Eder *et al*, 2002; Herbst *et al*, 2002a; Thomas *et al*, 2003). These studies administered daily endostatin doses of 15–600 mg m^−2^ day^−1^ by short intravenous infusion. No significant endostatin-related toxicity was noted. Endostatin displayed consistent linear pharmacokinetics with the area under the serum concentration–time curve reaching levels associated with activity in animal models, at doses of 300 mg m^−2^ day^−1^. No formal disease responses were seen although some evidence of antineoplastic activity was noted with one patient with metastatic pancreatic neuroendocrine tumour experiencing a minor response (Eder *et al*, 2002). However, these suggestions of antitumour activity did not appear to be dose-related and a subsequent phase II study of twice daily subcutaneous endostatin in metastatic neuroendocrine tumours only documented two minor responses in 41 patients (Kulke *et al*, 2003).

One problem for the phase I development of all antiangiogenic agents, which is exemplified by endostatin, is the difficulty in establishing the biologically effective dose (BED). All three phase I studies utilised radiological and biopsy-based indicators of tumour angiogenesis to assess BED. Thomas *et al* (2003) failed to demonstrate any significant changes by *in vivo* imaging with dynamic CT (marker of microvessel density), dynamic MRI (marker of tumour blood flow) or [^18^F]FDG PET (measure of tumour glucose metabolism). No changes in microvessel density, endothelial cell apoptosis, proliferation or vessel maturity were seen in paired pre- and 8-week post-treatment tumour biopsies from patients in this study although only eight sample pairs were available for analysis.

Herbst *et al* (2002b), however, in their study of 26 patients detected complex effects on tumour blood flow and biopsy biomarkers of tumour angiogenesis. Utilising [^15^O]H_2_O PET, they noted that blood flow decreased by an average of 20% with endostatin treatment by day 28 at the 180 mg m^−2^ day^−1^ dose level. Subtle changes in intratumoral glucose metabolism were also noted at higher endostatin doses. Changes in intratumoral blood flow only reached statistical significance however if patients treated at the 30 mg m^−2^ day^−1^ dose level were included. These patients did not have baseline PET imaging and so changes during cycle 2 instead of cycle 1 were used in the assessment of this cohort. Tumour biopsy analysis for microvessel density, endothelial cell apoptosis and nuclear localisation of HIF-1*α* as a marker of tumour hypoxia also suggested that endostatin was having an *in vivo* biological effect on human cancer (Davis *et al*, 2004). Intriguingly, statistical analysis using the quadratic polynomial model indicated that these effects may have a U-shaped relationship with dose, being most apparent around 250 mg m^−2^ day^−1^. While such exploratory analyses give us a potential insight into a BED for endostatin, they should be interpreted cautiously for several reasons. It is possible that the analysis employed was highly dependent on the range of doses used and may be distorted by the presence of outlying values. Seven patients (28%) with early progressive disease on study were excluded from these analyses owing to a lack of post-treatment biopsies and most importantly, these signs of biological activity were not translated into clinical activity. It is also impossible to rule out a contribution of tumour biology to the changes documented as no information is available on the natural history of these parameters in untreated malignancy.

## WHAT CAN BE LEARNED FROM THE ENDOSTATIN TRIALS?

Although the studies discussed above confirmed the safety of endostatin as a pharmacological agent, the lack of antineoplastic and antiangiogenic activity was disappointing. What lessons can be drawn from these studies for the future clinical development of endostatin and other endogenous angiogenesis inhibitors?

### Appropriate trial design, administration regimen and patient selection

Conventionally, most phase I studies are conducted in patients with bulky chemotherapy-resistant metastatic disease. Such patients are not ideal for the assessment of antiangiogenic agents, as not only do the tumours already have an established vasculature but the cancer cells are likely to have become tolerant of hypoxia and other apoptosis-inducing signals. It may be more appropriate, once toxicity data are available, to assess these agents in patients with minimal residual disease.

Endostatin was administered as short daily intravenous infusions in the published phase I studies. However, initial animal data were generated using the subcutaneous administration of a poorly soluble form of endostatin that is likely to have acted as a depot (O'Reilly *et al*, 1997) and subsequent work has shown that continuous infusion of endostatin is markedly more effective than bolus administration in animal models (Kisker *et al*, 2001). The most efficacious preclinical regimen should be adopted in clinical studies if feasible.

Tumour response may also be an inappropriate end point in phase II studies of endogenous angiogenesis inhibitors, as such molecules may only result in disease stabilisation. Trial designs assessing time to disease progression such as the randomised discontinuation model (Rosner *et al*, 2002) may be more sensitive to a biological effect.

### Clear understanding of antiangiogenic mechanism

At the time of the design of the endostatin trials, very little was known about endostatin's mechanisms of action. This clearly inhibited the scope of biomarker/translational research undertaken. The complexity of signalling pathways modulated by endostatin in endothelial cells *in vitro* means that no single biomarker can be proposed with certainty for investigation in future trials although assessment of *α*_5_*β*_1_ integrin expression and downstream signalling should be considered. The emerging evidence for a role of cell surface HSPGs in endostatin's function means that the use of heparin, particularly as a line-lock, may have abrogated its clinical activity. Such treatment was specifically contra-indicated in only one trial (Herbst *et al*, 2002a).

The clearly defined antiangiogenic mechanism for tumstatin indicates that assessment of PI3K–mTOR–4E-BP1 pathway in tumour endothelial cells should be included as a pharmacodynamic end point in any phase I tumstatin trials.

### Immunogenicity, intravascular proteolysis and a potential platelet sink

The administration of exogenous protein is associated with an immunogenic risk. Although no significant allergic reactions were seen with endostatin, many patients rapidly developed anti-endostatin IgG titres (Eder *et al*, 2002; Herbst *et al*, 2002a). Whether these antibodies are function-neutralising is not clear but their rapid induction is concerning.

Another immunological factor that may impinge on the clinical development of tumstatin is the localisation of the epitope recognised by the pathogenic auto-antibodies for Goodpasture's syndrome (GPS), a condition characterised by rapidly progressive glomerulonephritis and pulmonary haemorrhage, at the N-terminus of the tumstatin domain of *α*3 collagen IV (Hudson *et al*, 2003).

The potential clearance of circulating proteins by plasma and ECM proteases is a concern with all endogenous antiangiogenics. Although pharmacokinetic data were linear and analysis of serum-isolated endostatin in one phase I study (Thomas *et al*, 2003) did not demonstrate extensive proteolytic degradation, small shifts in mass spectrometry profiles were noted that could be consistent with limited terminal proteolytic cleavage. Of note, no *in vitro* activity could be demonstrated for re-isolated endostatin in any of the three studies.

In addition, platelets sequester many angiogenic regulators and have recently been shown to take up endostatin in a selective and quantifiable manner (Klement *et al*, 2004). This may indicate that the circulating platelet mass may need to become saturated with exogenously administered endostatin before it is able to target endothelial cells.

## OTHER POTENTIAL HURDLES

### Vasculogenic mimicry

Many established tumours have blood vessels lined in part or completely by tumour cells. In some cases, these cells express surface markers normally confined to endothelial cells. Tubular network formation by such cell lines is unaffected by endostatin treatment and other angiogenesis inhibitors (van der Schaft *et al*, 2004), indicating that the vascularity of some tumours may not be altered even if optimal inhibition of endothelial cell function is achieved *in vivo*. Some lung carcinomas also co-opt the host's existing alveolar capillary network. They are therefore less reliant on neoangiogenesis and unlikely to be sensitive to antiangiogenic therapies (Pezzella *et al*, 1997).

### Targeting an already overloaded system

Elevated serum endostatin concentration is seen in many untreated cancer patients and has been identified as an adverse prognostic factor. It is unclear whether these elevated endostatin levels are due to direct intratumoral production or a coordinated ‘host’ response to proangiogenic stimuli, in particular VEGF produced by the cancer (Feldman *et al*, 2002; Glenjen *et al*, 2002; Iizasa *et al*, 2004). What such findings do suggest however is that if this endogenous endostatin is in an active form, further increasing serum endostatin levels to pharmacological levels may not have a significant additional antiangiogenic effect as it is possible that in clinically manifest tumours, once the angiogenic switch has been tripped, raising the levels of endogenous antiangiogenic agents further is not very effective in resetting this. A further alternative explanation is that in effect these cancers are already at the top of the dose–response curve for endostatin and so have overcome its inhibitory effects. This possibility would have significant implications for the use of ECM proteolytic fragments as single agents in the therapeutic setting.

## FUTURE DIRECTIONS

### Rational combinations

Preclinical studies have demonstrated that the antitumour effects of endostatin are additive to radiotherapy (Shi *et al*, 2003) and synergistic with cytotoxic chemotherapy (Plum *et al*, 2003) in animal models. Importantly, no exacerbation of treatment-related toxicity was seen. Importantly, the recently reported preliminary results of a Chinese phase III trial appear to confirm these findings in man. The administration of a novel recombinant endostatin produced in *Escherichia coli* (Endostar™) in combination with chemotherapy in advanced non-small-cell lung cancer significantly increased response rates and time to progression when compared to chemotherapy alone (Sun *et al*, 2005). Publication of the full results is eagerly awaited.

More intriguingly, several studies have demonstrated synergy between endostatin and other antiangiogenic agents. Rational combinations determined by analysis of microarray data have been tested successfully in animal models. Cline *et al* (2002) noted markedly different gene expression profiles when endothelial cells were treated with TNP-470, a synthetic fumagillin analogue, or endostatin and subsequently showed synergistic activity with these agents against Lewis lung carcinoma xenografts. The results of Abdollahi *et al* (2004) indicated that endostatin downregulated many of the key mediators of VEGF signalling and notably, endostatin and SU5416, a small molecule tyrosine kinase inhibitor of KDR, are synergistic in inhibiting endothelial cell proliferation and the growth and vascularisation of tumour xenografts (Abdollahi *et al*, 2003). Tumours also grow more rapidly in double tumstatin/TSP-1 knockouts than in mice lacking only one of these molecules, suggesting a broader applicability of the concept of combined antiangiogenic therapy (Sund *et al*, 2005).

### Peptide fragments

One potential problem with the clinical development of antiangiogenic ECM proteolytic fragments is the requirement to synthesise large quantities of pharmaceutical grade protein. One way of reducing these manufacturing costs may be to identify component peptides from within these molecules that retain antiangiogenic activity. For example, a modified nonapeptide, ABT-510, derived from the second type-1 repeat of TSP-1 has shown promising preclinical and clinical antiangiogenic activity (Haviv *et al*, 2005). Notably, the antiangiogenic properties of tumstatin can be mimicked by a peptide encompassing amino acids 54–132 (Maeshima *et al*, 2001). The loss of the N-terminal domain of tumstatin in this peptide is potentially of clinical importance as this removes the epitope responsible for GPS.

Endostatin-derived peptides have also been investigated. However, the antiangiogenic activity has been shown to reside in different regions of the molecule by different groups. For instance, a peptide encompassing the secondary heparin-binding site was the minimal active domain in one *in vivo* study (Olsson *et al*, 2004) and the N-terminal 29 amino acids critical in another (Tjin Tham Sjin *et al*, 2005). While both peptides are antiangiogenic, it is not clear whether they function by the same intracellular effector pathways as each other or as parental endostatin.

## CONCLUSIONS

The identification of a series of cryptic proteolytic fragments of ECM components as endogenous angiogenesis inhibitors has shed new light on our understanding of the control of blood vessel development (Nyberg *et al*, 2005). It is intriguing that integrin-dependent signalling pathways have emerged as critical downstream mediators of the antiangiogenic properties of many of these fragments and that endostatin induces a profound, coordinated response in the angiogenic gene expression profile of endothelial cells (Abdollahi *et al*, 2004).

The discovery of these molecules has also opened up a significant new avenue in the development of antineoplastic agents. While the clinical development of endostatin as an antiangiogenic drug to date has been disappointing, it has enabled us to learn a number of lessons that could improve the strategy used to take other fragments, for instance tumstatin, through to the clinic. It is desirable that careful biological assays are conducted in a variety of systems and that a defined mechanism of action has been elucidated prior to clinical trials so that trial design can be targeted and appropriate candidate biomarkers can be selected for testing alongside conventional toxicity screening.

The ultimate future of these ECM-derived angiogenesis inhibitors may however lie in the development of rational combinations either with conventional antineoplastic therapy or other antiangiogenic agents.

## Figures and Tables

**Table 1:** Proteolytic fragments of ECM components with antiangiogenic properties

**Proteolytic fragment**	**ECM protein**	**Putative cell surface receptors**	**Possible mechanisms of action**
*Collagen chain (NC1 domain)*			
Arresten	*α*1 collagen IV	*α*1*β*1 integrin and HSPG	Interference with Ras–Shc–MAPK signalling
Canstatin	*α*2 collagen IV	*α*v*β*3 integrin	Downregulation of FLIP. Inhibition of PI3K/Akt signalling
Tumstatin	*α*3 collagen IV	*α*v*β*3 and *α*6*β*1 integrins	Inhibition of CAP-dependent protein synthesis
*α*6 NC1	*α*6 collagen IV	*α*v*β*3 integrin	ND
Endostatin	*α*1 collagen XVIII	*α*5*β*1 integrins and HSPGs	See text
Restin	*α*1 collagen XV	ND	ND
Vastatin	*α*1 collagen VIII	ND	ND
			
*Other ECM proteins*			
Anastellin	First type III repeat of fibronectin	ND	Inhibition of ERK signalling
Endorepellin	Perlecan	*α*2*β*1 integrin	Upregulation of PKA and FAK activity
TSP-1 fragments	Thrombospondin-1	CD36/ *β*1-integrins	Inhibition of PI3K
PEX	MMP-2	*α*v*β*3 integrin	Blocks cell surface activity of MMP-2

ECM=extracellular matrix; HSPG=heparan sulphate proteoglycan; MAPK=mitogen-activated protein kinase; PI3K=phosphatidylinositol-3 kinase; ND=not defined; ERK=extracellular signal-regulated kinase; FAK=focal adhesion kinase; PKA=protein kinase A; MMP-2=matrix metalloproteinase-2; Nyberg *et al* (2005) and references therein.

**Table 2:** Phenotypic manifestations associated with the deletion of ECM proteins

**ECM protein**	**Associated fragment**	**Phenotype**
Collagen XVIII	Endostatin	Knobloch's syndrome – myopia, vitreoretinal degeneration, occipital encephalocoele Knockout mouse – abnormal ocular vasculature
Collagen XV	Restin	Knockout mouse – skeletal myopathy, abnormal cardiac and skeletal muscle capillaries
Collagen IV*α*3	Tumstatin	Alport's syndrome – haematuria, proteinuria, renal failure, sensorineural deafness Knockout mouse – renal failure secondary to glomerular basement membrane abnormalities, more rapid growth and increased vascularisation of tumour xenografts
Perlecan	Endorepellin	Knockout mouse – embryonic death, intrapericardial haemorrhage and cardiac developmental abnormalities

ECM=extracellular matrix; Hudson *et al* (2003), Marneros and Olsen (2005) and Nyberg *et al* (2005).
